# Optoacoustic microscopy at multiple discrete frequencies

**DOI:** 10.1038/s41377-018-0101-2

**Published:** 2018-12-19

**Authors:** Stephan Kellnberger, Dominik Soliman, George J. Tserevelakis, Markus Seeger, Hong Yang, Angelos Karlas, Ludwig Prade, Murad Omar, Vasilis Ntziachristos

**Affiliations:** 1 Institute of Biological and Medical Imaging (IBMI), Helmholtz Zentrum München, Ingolstädter Landstr. 1, 85764 Neuherberg, Germany; 2 Chair of Biological Imaging (CBI) and Center for Translational Cancer Research (TranslaTUM), Technical University Munich, Einsteinstraße 25, 81675 München, Germany; 3 Cardiovascular Research Center, Cardiology Division, Massachusetts General Hospital, Harvard Medical School, Boston, MA 02114 USA; 4 Xidian University, South Taibai Road 02, 710071 Xi’an, Shaanxi China

## Abstract

Optoacoustic (photoacoustic) sensing employs illumination of transient energy and is typically implemented in the time domain using nanosecond photon pulses. However, the generation of high-energy short photon pulses requires complex laser technology that imposes a low pulse repetition frequency (PRF) and limits the number of wavelengths that are concurrently available for spectral imaging. To avoid the limitations of working in the time domain, we have developed frequency-domain optoacoustic microscopy (FDOM), in which light intensity is modulated at multiple discrete frequencies. We integrated FDOM into a hybrid system with multiphoton microscopy, and we examine the relationship between image formation and modulation frequency, showcase high-fidelity images with increasing numbers of modulation frequencies from phantoms and in vivo, and identify a redundancy in optoacoustic measurements performed at multiple frequencies. We demonstrate that due to high repetition rates, FDOM achieves signal-to-noise ratios similar to those obtained by time-domain methods, using commonly available laser diodes. Moreover, we experimentally confirm various advantages of the frequency-domain implementation at discrete modulation frequencies, including concurrent illumination at two wavelengths that are carried out at different modulation frequencies as well as flow measurements in microfluidic chips and in vivo based on the optoacoustic Doppler effect. Furthermore, we discuss how FDOM redefines possibilities for optoacoustic imaging by capitalizing on the advantages of working in the frequency domain.

## Bioimaging: Low-cost lasers at discrete modulation frequencies get the blood flowing

Using continuous wave (CW) lasers to power optoacoustic imaging may help researchers capture blood flow and micro-structures inside living tissue. By stimulating the emission of ultrasound waves deep inside animal or human subjects with short bursts of laser light, optoacoustic (photoacoustic) microscopes produce 3D images of cellular structures noninvasively. Vasilis Ntziachristos and colleagues from the Technical University Munich and Helmholtz Center Munich in Germany report that high-fidelity optoacoustic images can now be obtained using cost-efficient lasers with light intensity modulated at discrete frequencies. The use of multiple discrete frequencies is critical for producing high fidelity images. The authors demonstrated imaging of structural details from fish and mouse tissue microvasculature, by superimposing structural details that only appear at certain frequencies. Tracking of frequency shifts due to the optoacoustic Doppler effect further enabled the identification of blood flow speed in tissue microvasculature.

## Introduction

Most optoacoustic implementations operate in the time domain (TD) using laser pulses that last 1–100 ns^1,2^. The ultra-short pulses stimulate the emission of broadband ultrasonic waves, which are collected in the microsecond range and utilized to form optoacoustic images^3,4^. Nanosecond light pulses of sufficient energy generate strong energy gradients, which are necessary for providing good signal-to-noise ratios (SNRs) but require laser technology that is complex, expensive, and cannot simultaneously deliver multiple wavelengths.

TD operation has been a common first step in the experimental introduction of various modalities, including optical coherence tomography and nuclear magnetic resonance. However, due to the advantages of frequency-domain methods, mainstream implementations of these modalities have been primarily considered in the frequency domain (FD)^5–7^. In optoacoustics, FD imaging relies on an intensity-modulated continuous energy stream instead of photon pulses, which are used in TD implementations. The use of intensity-modulated light enables (1) higher repetition rates, (2) information encoding at various frequencies (e.g., frequency encoding at multiple wavelengths), and (3) the use of cost-effective hardware. However, the application of FD optoacoustic imaging at a single frequency failed to generate high-fidelity images of tissues^8^ since images consist of multiple spatial frequencies, but demonstrated the spectrometry potential for sensing oxygen saturation using two wavelengths^9^. Frequency-encoded time-of-flight measurements based on light of frequency-varying chirp modulation have also been considered for optoacoustic imaging at one or two wavelengths^10–14^. In this case, detection is performed in the TD based on cross-correlation of excitation and detection waveforms to retrieve the times of flight of the detected ultrasound waves. Therefore, optoacoustic imaging implementations have so far been based on techniques that detect signals in the TD (photon pulses and chirp excitation) or only scan a single frequency in the FD.

In this work, we investigated a fundamentally different approach for optoacoustic imaging by employing continuous-wave (CW) light with the intensity being modulated at multiple discrete frequencies. Detection was based on time-independent homodyne demodulation techniques and sampled signals in a mixed real-space/k-space domain. We hypothesized that detection of the amplitude and phase of ultrasound waves excited at multiple discrete modulation frequencies could broadly capture the spatial frequencies of the imaged object, thereby leading to high-fidelity images. To examine this hypothesis, we developed a multi-wavelength FD optoacoustic microscopy (FDOM) system that operates in the frequency range of 5–50 MHz. FDOM was implemented with multiphoton microscopy as a hybrid modality. Using modulation frequencies up to two orders of magnitude greater than the repetition rates of TD optoacoustic microscopes, we performed two- and three-dimensional imaging based on ultrasound amplitude and phase measurements at multiple modulation frequencies. We interrogated the relationship between modulation frequency and image fidelity and examined the SNR achieved via TD approaches. We realized intravital optoacoustic imaging in the FD for the first time based on concurrent illumination with two wavelengths. We also discovered that the use of discrete frequencies enables the measurement of optoacoustic Doppler shifts, thereby enabling flow observations both in a microfluidic flow chamber and in tissue microvasculature in vivo.

## Results

### FDOM and multiphoton microscopy

**Fig. 1:**
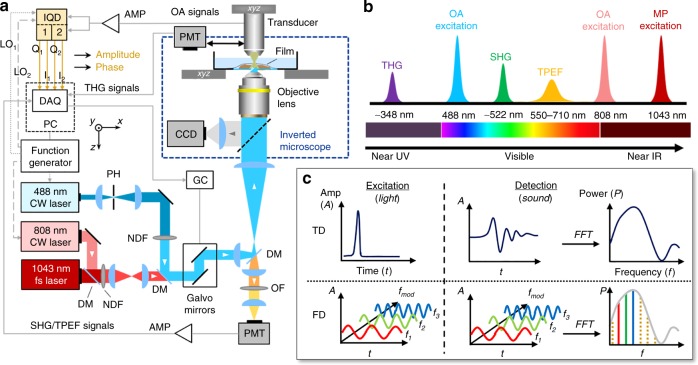
Schematic representation of the hybrid microscopy system, which consists of a subsystem for dual-wavelength optoacoustic microscopy at 488 nm and 808 nm co-aligned with a subsystem for multiphoton microscopy at 1043 nm. **a** AMP amplifier, CCD bright-field camera, DAQ data acquisition card, DM dichroic mirror, GC galvanometric mirror controller, IQD IQ demodulator, LO1 local oscillator 1, LO2 local oscillator 2, NDF neutral density filters, OA optoacoustic, OF optical filter, PC personal computer, PH pinhole, PMT photomultiplier tube, SHG second-harmonic generation, THG third-harmonic generation, TPEF two-photon excitation fluorescence, *xyz* motorized stages. **b** The spectrum of the excitation and detection wavelengths in hybrid FDOM/multiphoton (MP) imaging. **c** Schematic comparison between time-domain (TD) optoacoustic microscopy, which uses short pulses of light, and frequency-domain (FD) optoacoustic microscopy, which is based on a modulated laser intensity at multiple discrete frequencies (the figure is only for representation purposes; it is neither scalable nor quantitative)

FDOM excitation utilizes the same optical path as multiphoton microscopy (Fig. 1a); each modality operates in distinct spectral bands (Fig. 1b). FDOM scans (of an area of ~300 × 300 pixels) were performed at 488 nm and 808 nm using intensity-modulated CW laser diodes. Light was coupled to an air immersion objective lens (NA 0.45) via a galvanometric scanner that enabled raster scanning (Fig. 1a; see Materials and methods for details). The scanning time was ~0.6 s using a fluence of ~0.5 mJ·cm^−2^ per wavelength. The wavelengths of 488 nm and 808 nm were loaded on separate frequencies that were modulated over a range of 5 to 50 MHz, typically in 5-MHz steps (Fig. 1c; see Materials and methods and Supplementary Fig. S1 for details). This provided concurrent multicolor illumination, which is unique to FD methods.

Optoacoustic signals were detected using a spherically focused piezoelectric transducer with a central frequency of 75 MHz and a −12-dB bandwidth of 117 MHz (see Supplementary Fig. S2 for details). The transducer was aligned to the focus of the illumination objective in transmission mode in a defocused position to cover the scanned area. Acoustic focusing was realized using a glass lens (3-mm thick) that was fitted in front of the transducer. The amplitude and phase of the generated optoacoustic signals were resolved via homodyne-based IQ demodulation in real time and recorded using an analog-to-digital converter.

Co-localized multiphoton microscopy was realized using femtosecond laser excitation at 1043 nm, which was coupled to the FDOM beam path using a dichroic mirror and directed through the same objective lens that was used for FDOM illumination (Fig. 1a). Second harmonic generation (SHG) and two-photon excitation fluorescence (TPEF) signals passed through a dichroic mirror and were detected in the backward direction by a photomultiplier tube (PMT), while third harmonic generation (THG) signals were recorded in the transmission direction using another PMT that replaced the transducer.

### Frequency-space characterization

**Fig. 2:**
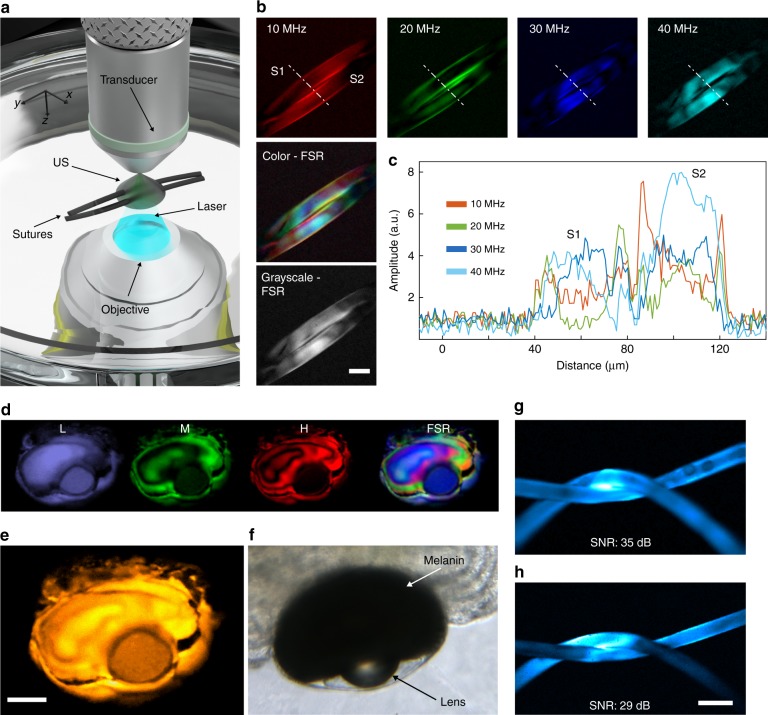
Single-wavelength FDOM imaging of a suture phantom and ex vivo zebrafish samples. **a** A schematic illustration of the scanning of two crossing sutures. **b** Color-coded FDOM images of two 50-µm sutures, which are based on illumination at 488 nm and modulation frequencies of 10, 20, 30, and 40 MHz. The color frequency-space representation (FSR) superimposes the contributions by each modulation frequency. The grayscale FSR image based on four frequencies shows the final image. Scale bar, 50 µm. **c** Cross-sectional profile of the dashed line shown in panel b, which compares the contrasts revealed by the various modulation frequencies. **d** *Ex vivo* imaging of a zebrafish larva eyeball. The purple image was reconstructed using low (L) frequencies (10, 15, and 20 MHz); the green image using middle (M) frequencies (25, 30, and 35 MHz); and the red image using high (H) frequencies (40, 45, and 50 MHz). The color-coded overlay of all frequencies (FSR, 10 to 50 MHz) highlights the contribution of each spectral region. **e** Orange color depicts the amplitude sum for the nine employed modulation frequencies. Scale bar, 100 µm. **f** A bright-field image of a zebrafish eye, validating the fidelity of FDOM images. **g** A comparison of the signal-to-noise ratios (SNRs) of images of two crossing sutures (40 µm diameter) obtained via FD and TD optoacoustic microscopy. The FDOM image yielded an SNR of ~35 dB. **h** Under similar experimental settings, TD microscopy resulted in an SNR of ~29 dB. Scale bar, 100 µm

To identify the characteristics of FD optoacoustic image formation, we imaged a pair of crossed sutures (50 μm in diameter) in water (Fig. 2a and Supplementary Fig. S3) at wavelengths of 488 nm (Fig. 2b) and 808 nm (not shown) at discrete modulation frequencies. Different modulation frequencies excited and captured different spatial frequency components (equivalent to k-space sampling; see the image formation below, Fig. 2b, c, and Supplementary Fig. S4 for details). Each panel in Fig. 2b is an image of the object at the selected modulation frequency. The superposition of various frequency contributions, which is depicted in the color image in Fig. 2b, shows the image information that is carried by each modulation frequency.

Summing the spatially obtained amplitudes of single excitation frequencies enabled multiple spatial frequencies to be superimposed in a single overlay image, which demonstrates that different frequencies capture complementary information in the image and improve the image quality over images at individual modulation frequencies (Fig. 2b). To interrogate whether spatial frequencies can be extracted from more complex structures than sutures, we imaged the eye of a 5-day-old wild-type zebrafish larva ex vivo using nine modulation frequencies that span 10–50 MHz in 5-MHz steps.

Ripples were clearly observed, and their appearance depended on the modulation frequency band that was employed (Fig. 2d). Color-coded summation of images at various frequencies revealed complementarity among the observed ripples (Fig. 2d–f), thereby leading to a better representation of the sample than when using individual frequency bands. Hybrid FDOM-TPEF imaging of an 8-day-old transgenic Casper-type zebrafish embryo ex vivo that expressed green fluorescent protein (GFP) in its nervous system (*Tg(brn3:GFP)*) demonstrated the inherent image co-registration between the optoacoustic and multiphoton systems (Supplementary Fig. S5).

### SNR analysis

As a critical next study, an SNR comparison between FDOM and conventional TD optoacoustic imaging was performed using a phantom of black sutures (40 µm in diameter) embedded in scattering agar. FDOM was performed at a wavelength of 488 nm, a fluence of ~100 W·cm^−2^ at the sample, and nine modulation frequencies that ranged from 10 to 50 MHz (Fig. 2g, h). TD imaging was performed using a diode-pumped solid-state laser at 532 nm, a pulse width of 1.4 ns, and a pulse energy of ~10 nJ at the sample, which are typical parameters in TD optoacoustic microscopy^15^. The same ultrasound transducer was used for FDOM and TD microscopy. Assuming identical acquisition times for FD and TD imaging, we obtained SNRs of ~35 dB for FDOM and ~29 dB for TD microscopy from images Fig. 2g and h, which we corroborated via theoretical SNR calculations (see Materials and methods for details). These results are computed for an implementation that assumes a single TD measurement at a repetition rate of 20 kHz vs. 100 FD averages at 240 kS·s^−1^ and demonstrate that FDOM, because of its significantly higher repetition rate, can achieve a similar SNR compared with TD optoacoustic microscopy. Advantageously, in contrast to TD systems, in which the scan speed is limited by both the SNR and the time of flight of ultrasound measurements from the object to the detector, the temporal resolution of FDOM is primarily limited by the SNR. Naturally, these SNR findings will vary according to the laser energy and power employed in our experiments as well as the detection bandwidths of the data acquisition hardware used in TD and FD, respectively. Our current FDOM operates with a sub-optimal sampling rate of 240 kS·s^−1^; however, the sampling speed can be increased by using higher-end detection systems. Therefore, the measurements described herein could serve as a reference and as a basis for computing the relationship between TD and FD SNRs under various illumination conditions.

### Image formation

Superimposing images obtained at different modulation frequencies represents a simplistic image formation approach and demonstrates the complementarity of data obtained at single frequencies (Fig. 2 and Supplementary Fig. S6a). More generally, multi-frequency amplitude and phase data can be processed for three-dimensional image reconstruction using the Fourier transform based on two approaches: frequency-space representation (FSR) and time-space representation (TSR). The FSR arranges data in a three-dimensional matrix, which is denoted as *S(x, y, f)*, in which the *x*- and *y*-axes correspond to the spatial scanning points and the *f*-axis corresponds to a vector that contains the measurement (amplitude and phase) at each modulation frequency. The TSR arranges data in a three-dimensional matrix, which is denoted as *S*_*TSR*_ *(x, y, t)*, in which the *x*- and *y*-axes also correspond to the *x–y* scan pattern and the *t*-axis is a time-dependent signal obtained via the inverse Fourier transform (IFT), namely, ${\boldsymbol{S}}\left( {x,y,f} \right)\mathop{\longrightarrow}\limits^{{IFT}}S{}_{TSR}\left( {x,y,t} \right)$, of the collected FD signals (Supplementary Fig. S6b).

TSR data can be processed by applying the Hilbert transform and used to generate an image via conventional TD optoacoustic inverse algorithms. In contrast, FSR imaging can be performed based on inversion and k-space sampling, e.g., by applying synthetic aperture diffraction tomography or the Stolt’s *f-k* migration algorithm^16,17^.

In the most trivial case of single modulation frequencies *f* = 1, 2, … *n*, where *n* is the total number of optical modulation frequencies, the FSR of the recorded data, namely, *S(x, y, f)*, can be regarded as a projection of each frequency component *f* onto the two-dimensional map *R(x, y)* and the resulting FD optoacoustic image represents a summation of all modulation frequencies: ${\mathrm{Recon}}_{FSR}\left( {x,y,\mathop {\sum}\nolimits_{i = 1}^n f } \right)$ (Supplementary Fig. S6a). The results of FSR-based reconstruction are similar to those that were obtained with TSR (Supplementary Fig. S7); however, the calculations are faster, as no data inversion is involved in image reconstruction. Therefore, all subsequent imaging reconstructions were performed via the FSR approach. In general, computational acceleration is a feature of systems that use FD-based data processing (Telenkov et al.)^18^.

### In vivo imaging

**Fig. 3:**
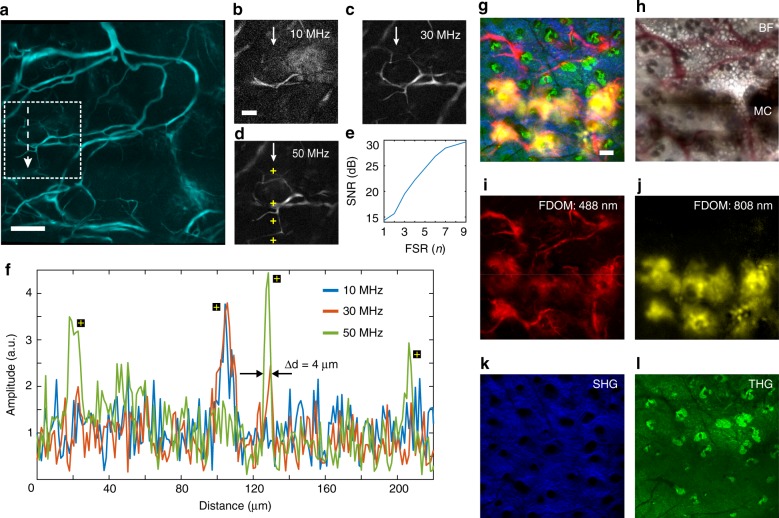
Single- and dual-wavelength FDOM imaging of a mouse ear in vivo. **a** FDOM imaging at 488  nm. Cyan color represents the reconstructed image, namely, ${\mathrm{Recon}}_{FSR}\left( {x,y,\mathop {\sum}\nolimits_{i = 1}^n f } \right)$, from nine equally spaced frequencies in the range of 10 to 50 MHz. Scale bar, 150 μm. **b**–**d** Individual images obtained at modulation frequencies of 10, 30, and 50 MHz, which depict the structures in the dashed box in panel **a**. Scale bar, 50 μm. **e** SNR as a function of *n* frequencies that were used for FSR reconstruction. An asymptotic improvement is observed for *n* > 8 discrete frequencies. **f** A profile view of the dashed box in panel **a**, which is delineated by a white dashed arrow. It demonstrates the relationship between modulation frequency and imaging resolution. Yellow crosses highlight the imaging resolution as a function of the modulation frequency: faster modulation (50 MHz) can clearly resolve small structures, even down to 4 µm, while slower modulation (10 MHz) cannot. **g**–**l** Hybrid FDOM/multiphoton imaging of a mouse ear following the injection of melanoma cells. **g** An overlay image that was obtained using four label-free microscopy modalities: FDOM at 488 nm and 808 nm, SHG at 522 nm, and THG at 348 nm. **h** A bright-field image validating the results that were obtained via hybrid microscopy; MC, melanoma cells. **i** FDOM imaging at 488 nm showing vasculature and melanoma cells. **j** An FDOM image at 808 nm that shows B16F10 melanoma cells injected in the mouse ear. **k** An SHG image showing the collagen distribution in the epidermis. **l** A THG image that shows the tissue morphology; predominantly keratinocytes and hair follicles

We assessed the ability of FDOM to image tissues in vivo by visualizing the ear of an anesthetized mouse (Hsd athymic nude foxn1) at a wavelength of 488 nm using nine modulation frequencies in the range of 10 to 50 MHz at 5-MHz steps. All imaging experiments were performed with laser powers that are well below the safety standards defined by the American National Standards Institute (ANSI)^19^. A field of view (FOV) of 360 × 360 µm² was scanned in steps of 1.2 µm. Adjacent FOVs were stitched together to form a total area of 960 × 960 µm², in which microvasculature of diameters that ranged from 4 µm (small capillaries) to 33 µm was resolved (Fig. 3a). Observation of images that were reconstructed at single modulation frequencies (Fig. 3b–d), which correspond to the area marked with a dotted box in Fig. 3a, revealed a relationship between the modulation frequency and the sizes of the optical absorbers in the image, which is similar to our findings in Fig. 2b. We found that single-frequency images only capture a subset of the image features of the imaged object and fail to reconstruct a high-fidelity image that contains all structures in an object (see also Supplementary Fig. S4). Specifically, microscopic details such as small vessels were clearly visible in the 50-MHz image (Fig. 3d), but not in images obtained at 10 and 30 MHz (Fig. 3b, c). Larger vessels appeared more homogeneous in images obtained at 10 and 30 MHz compared with 50 MHz, based on line profiling through the image (Fig. 3f). This measurement also reveals the relationship between the spatial frequency of the object and the optical modulation frequency, which demonstrates that multiple modulation frequencies that are matched to the spatial frequencies of the imaged object are needed to resolve an artifact-free image. As expected, the SNR of the image increased from ~14 dB at a single frequency to ~30 dB at nine frequencies (Fig. 3e and Supplementary Fig. S8). We did not explore the use of more than nine frequencies because the SNR improved by only ~2 dB between eight and nine frequencies, as shown in the image comparison in Supplementary Fig. S8, which experimentally supported the numerical findings of previous simulation studies that used optoacoustic amplitude and phase measurements^20^.

Next, we performed in vivo imaging of a mouse ear that contained B16F10 murine metastatic melanoma cells (see Materials and methods for details) via concurrent excitation at two wavelengths (488 nm and 808 nm) that were encoded at separate modulation frequencies, which is impossible in TD optoacoustics. Imaging at 488 nm is sensitive to blood vessels owing to the high absorption of hemoglobin^21^, while the imaged cell line B16F10 is responsive to near-infrared wavelengths^22^. We expected that B16F10 cells would be visible by FDOM at 488 nm and 808 nm because of the flat optical absorption spectrum of the contained melanin, whereas the vasculature would be nearly invisible at 808 nm because of the weak hemoglobin absorption of near-infrared light. The two wavelengths were separated in the Fourier space (Supplementary Fig. S1). Co-registered SHG, THG, and dual-wavelength FDOM images showed excellent label-free contrast (Fig. 3g–l), thereby enabling the differentiation of vascularization, which was revealed by 488-nm FDOM (Fig. 3i); melanoma cells, which were revealed by 488- and 808-nm FDOM (Fig. 3i, j); collagen fibers, which were shown by SHG at ~522 nm (Fig. 3k); and hair follicles and keratinocytes, which were shown by THG at ~348 nm (Fig. 3l). The total FOV that was obtained after stitching adjacent areas was 1220 × 1220 μm^2^ with ~2-μm pixel size for FDOM and 0.8-μm pixel size for the multiphoton modalities (Fig. 3g). Hybrid imaging results were validated via bright-field (BF) microscopy (Fig. 3h).

### Doppler flow

**Fig. 4:**
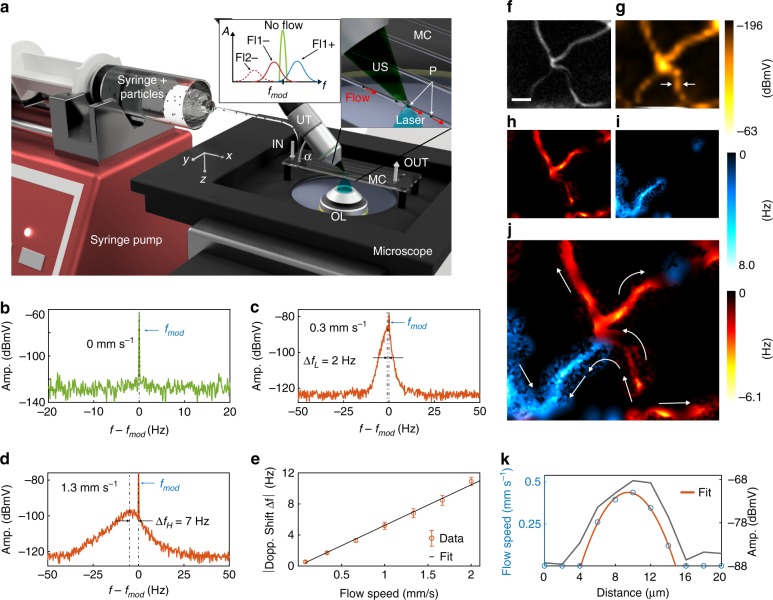
Optoacoustic imaging of microcirculatory blood flow in a mouse ear in vivo. **a** A scheme of the µDoppler detection set-up. FL1^−^ flow 1 away from the US sensor, FL2^−^ flow 2 away from the US sensor (FL2^−^ < FL1^−^), FL1^+^ flow 1 toward the US sensor, IN flow direction into the chip, MC microfluidic chip, OL objective lens, P particles, US ultrasound, UT ultrasound transducer, *f*_*mod*_ modulation frequency, OUT flow direction out of the chip. The close-up views illustrate the experimental detection of particles moving away from the ultrasound sensor, which is equivalent to **a** Doppler red shift. **b**-**d** Averaged frequency spectra acquired at flow speeds of 0 mm·s^−1^ (green), 0.3 mm·s^−1^ (red), or 1.3 mm·s^−1^ (red). The latter two flow speeds show respective red shifts of 2 Hz and 7 Hz from the modulation frequency because particles are flowing away from the transducer. **e** Doppler shifts measured from carbon particles as a function of the flow speed in a microfluidic chip. The black line shows a linear fit to the data. **f** A maximum intensity projection of a region of interest (ROI) of size 160 × 160 µm² in the mouse ear, which shows micro-vascularization. Scale bar, 30 µm. **g** A Doppler FDOM-flow map that was recorded in the same ROI, showing a peak amplitude of the recorded flow in the blood vessels. **h**, **i** A blend and an overlay of the Doppler flow map g and the optoacoustic image f, which show peak amplitudes as Doppler red and blue shifts relative to the transducer position. **j** An overlay of Doppler red- and blue-shift maps on the galvanometric scan in panel f. White arrows indicate the inferred directions of blood flow in various vessels. **k** A profile scan across a single capillary at the position indicated by the white arrows in the galvanometric scan in panel g. The red line represents a parabolic fit to the recorded Doppler shift data with a maximum blood flow speed of 0.44 mm·s^−1^. The gray solid curve shows the peak amplitudes at each measurement position

A unique feature of the FD implementation described herein is the availability of distinct modulation frequencies, which can be used, in principle, for direct flow recordings based on the optoacoustic Doppler effect, as compared with TD optoacoustic systems, which require indirect measurements that are mostly based on time-correlation methods. To experimentally examine this possibility for the first time in vivo, we performed FD micro-Doppler (µDoppler) measurements by placing the ultrasound detector at an angle *α* of ~55° with respect to the imaging plane (Fig. 4a).

As a first validation study, we analyzed black carbon particles (2–12 μm in diameter) circulating at varying flow speeds in a microfluidic chip with a channel cross-section of size 1 mm × 200 µm that was connected to a syringe pump (see Materials and methods for details). Ten consecutive measurements were collected at flow speeds between 0.1 and 2 mm·s^−1^ using a modulation frequency of *f*_*mod*_ = 15 MHz. The averaged frequency spectra recorded at flow speeds of 0, 0.3, and 1.3 mm·s^−1^ (Fig. 4b–d) exhibited a central peak (*f*_*mod*_), which corresponded to the modulation frequency that was employed and originated from non-moving objects and a spectral width that increased with the flow speed. Speeds of 1.3 mm·s^−1^ resulted in a Doppler shift of *Δf*_*H*_ *=* 7 Hz, whereas speeds of 0.3 mm·s^−1^ yielded a shift of *Δf*_*L*_ = 2 Hz. Spectral broadening can be attributed to two main factors: the finite size of the spherically focused transducer, which averages Doppler shifts at varying angles along its active element surface, and the presence of flowing absorbers traveling at different flow speeds through the channel^23^. The observed frequency spectrum shifts toward lower frequencies than *f*_*mod*_ (red shift) for particles flowing away from the transducer and toward higher frequencies than *f*_*mod*_ (blue shift) for particles moving toward the transducer. A plot of the average Doppler shifts as functions of the flow speed revealed a linear trend (*R*² = 0.996, Fig. 4e), which was used as a calibration curve for in vivo µDoppler FDOM experiments in a mouse ear. Via equation (3) (see Materials and methods), we experimentally determined the Doppler angle to be 58 ± 1°, which closely matched the assumed transducer angle of ~55° that was chosen for the µDoppler experiments. The integration time per scan position for all Doppler flow experiments in this work was ~8 s.

Then, µDoppler FDOM was employed to generate a map of the microcirculatory blood flow and a flow profile in vivo in a mouse ear. In these experiments, a region of the ear that measured 160 × 160 µm² was scanned under the same conditions as in Fig. 3a to obtain a high-resolution morphological image (Fig. 4f). Next, a Doppler scan was performed at 42 MHz within ~55 min in the same region of interest, thereby generating a Doppler shift flow map (Fig. 4g). Overlaying and blending confirmed a spatial congruence between the Doppler scans and the optoacoustic image of the mouse ear. Color coding further demonstrates the flow direction relative to the transducer, as shown in Fig. 4h, i. Flow analysis results for the overlay image (Fig. 4j) are marked with white arrows and demonstrate that vessels that apparently belong to the same vessel structure in the morphological image (Fig. 4f) in fact represent different vascular structures, as revealed by the color-coded flow analysis.

Cross-sectional flow profiling in 2-µm steps across the capillary highlighted by white arrows in Fig. 4g revealed a gradually increasing flow speed from the vessel edge to its core (Fig. 4k). A parabolic fit to the Doppler shift values (*R*² = 0.997) suggested a maximum flow speed of ~0.44 mm·s^−1^.

## Discussion

The main advantages of FD methods over TD approaches have already been explored in diverse applications ranging from telecommunications and radar technology to magnetic resonance imaging systems. In the present study, we introduce FD optoacoustics at multiple distinct frequencies using inexpensive CW laser diode illumination and explore the merits of this approach for intravital microcopy. Laser intensity modulation with frequency sweeps in the low MHz range can generate depth-profilometric images^12,18^. In this work, we discover that the use of multiple modulation frequencies is essential for achieving high image fidelity. We accurately capture various spatial frequencies, even when a focused laser beam is employed. We find that by using more frequencies, we can better capture image features; however, we also observe an asymptotic improvement when using more than eight frequencies, which is similar to the result obtained from our investigations of the numerical simulations in Mohajerani et al.^20^. This finding implies that conventional TD optoacoustic approaches based on broadband stimulation substantially oversample frequencies, thereby demonstrating an information acquisition redundancy.

Typically, spectral optoacoustic imaging in the TD is challenging due to the limitations of ultra-fast pulsed sources. Tunable pulsed lasers, including dye lasers and optical parametric oscillators that require Q-switched pump sources, are expensive and can illuminate at only one wavelength at a time. Single-wavelength pulsed lasers are less expensive and can be used in combination to provide near-simultaneous illumination at multiple wavelengths; however, few visible and near-infrared wavelengths are currently available. Only Nd:YAG lasers, which emit at 1064 nm, and the frequency-doubled variant, which emits at 532 nm, are commercially available. As we demonstrate in the present study, modulating the light source using CW lasers can make a wider range of wavelengths available for use. We further show that the wavelengths can be frequency-encoded in a process that is unique to FD, thereby enabling simultaneous illumination at multiple wavelengths. FDOM features a high repetition rate and induces narrowband optoacoustic signals^10,11^, thereby enabling cost-efficient and low-noise homodyne detection using simple analog-to-digital converters.

The combination of optoacoustic and optical microscopy enabled label-free visualization of several tissue features, which complements conventional interrogations that rely on fluorescence-based contrast and thereby offers multi-parametric biological readings^15,24^. Combining FDOM and various multiphoton modalities in a single setup enabled us to visualize soft tissue structures, such as micron-sized vasculature, melanoma cells, collagen, and keratinocytes in co-registered images.

Time-domain flow sensing approaches are indirect as they mostly rely on correlation-based methods. The ability of FDOM to exploit discrete frequencies enabled us to perform the first optoacoustic Doppler measurements of microcirculatory blood flow in vivo using lock-in detection. Previously, the optoacoustic Doppler effect has been used to track the flow of micrometer-sized particles only in phantoms^23,25,26^. FD-based Doppler measurements may provide a faster and more robust alternative to TD-based optoacoustic label-free blood flow measurements. This is because TD-based flow measurements require a sequential analysis of signals acquired from each scanning position via cross-correlation techniques^27,28^, which is not a real-time process and requires substantial computing time and memory. In addition, TD-based blood flow quantification requires knowledge of the optical focus size inside the vessel^28^.

In summary, FDOM is the first demonstration of time-independent optoacoustic signal detection and demodulation. Amplitude and phase signals are captured at multiple spatial frequencies of the imaged object. FDOM capitalizes on the advantages of FD methods, including inexpensive light sources with broad wavelength availability, demodulation-based detection, simultaneous multi-wavelength illumination, and direct Doppler-based flow measurements. To increase the performance of the system, several improvements are planned, such as simultaneous frequency modulation to support real-time applications. Future studies with our imaging system will focus on a quantitative evaluation of the modulation frequencies and imaging depth as well as increasing the resolution.

## Materials and methods

### Animal procedures

All zebrafish and mouse procedures were approved by the District Government of Upper Bavaria. Nude athymic mice (Foxn1, HSD) were used for FDOM imaging and µDoppler FDOM experiments. For hybrid imaging, B16F10 melanoma cells were injected between the skin and the cartilage on the dorsal side of the mouse ear 24 h prior to the FDOM experiment. During in vivo experiments, mice with injected B16F10 cells were imaged following anesthesia with 2% isoflurane; body temperature was maintained using a heat lamp and eye ointment was applied. During ex vivo experiments, inbred-strain CD1 mice (Charles River) were killed and their ears were excised prior to the experiment (see Supplementary Fig. S9). Eight-day-old Casper-type zebrafish that expressed GFP in their nervous systems (*TG(brn3:GFP)*) were imaged ex vivo using a customized sample holder.

### FDOM setup

Illumination was provided by two CW diode lasers (A350, Omicron-Laserage, Rodgau, Germany) that emitted light at 488 nm and 808 nm with output powers of ~200 mW and ~140 mW, respectively. Two function generators (DG5252, Rigol, Beijing, China) modulated the current of the laser diodes using a sinusoidal waveform within a frequency range of 10 to 50 MHz, thereby enabling a spurious-free dynamic range of more than 11 dB (for more details, see Supplementary Fig. S10). The intensity-modulated laser beam of the 808-nm laser was initially guided through an optical multimode fiber, whereas the 488-nm laser emitted a beam into free space. Both lasers were collimated, co-aligned using a long-pass dichroic mirror (DMLP650, Thorlabs, Newton, NJ, USA) and attenuated to the desired level using a neutral density filter set. High-reflectance mirrors directed both beams onto high-precision galvanometric mirrors (6215 H, Cambridge Technology, Bedford, MA, USA), which performed fast raster scanning (on the order of 1 Hz per image) of the optical focus across the transverse imaging plane within the sample.

After passing through the scanning system, the beams entered a telescope that consisted of two plano-convex lenses in a typical 4*f*-configuration; this expanded the beams approximately six-fold until they completely filled the back aperture of the objective lens. The expanded beams were coupled to a modified inverted microscope (AxioObserver.D1, Zeiss, Jena, Germany), where they were focused onto the sample using an objective lens (Plan Apochromat 10 × , Zeiss; air immersion, NA 0.45). Samples were placed in a water-filled Petri dish with a standard 170-μm coverslip attached to the bottom to ensure optimum focusing. The Petri dish was attached to a holder that was mounted on a high-precision motorized *xyz-*stage (MLS203–2 and MZS500-E, Thorlabs), with which the sample was positioned prior to optoacoustic imaging.

For in vivo mouse experiments, we used a 3D-printed mouse holder that was mounted on the sample-scanning *xyz*-stage and enabled the scanning of the mouse ear that was attached to a 170-µm coverslip. The ear was sandwiched between the coverslip below and a plastic film above. The film frame exerted slight pressure on the ear, thereby flattening it against the coverslip. Acoustic coupling was provided by ultrasound gel that was inserted between the ear and the plastic film and by water between the film and the transducer.

We used a spherically focused 75-MHz ultrasound transducer (SONAXIS, Besancon, France; focal distance, 3 mm; *F/D*, ~1) for ex vivo zebrafish imaging, in vivo mouse imaging, and Doppler flow experiments. The transducer was immersed in water above the sample in transmission mode, and the configuration was defocused by 500 to 600 µm relative to the laser beam focus to ensure an adequate acoustic detection field without losing significant spatial sensitivity of the acoustic sensor^29^. The transducer was positioned using a set of high-precision motorized stages (M-683.2U4 and M-501.1DG, PI, Karlsruhe, Germany).

Optoacoustic signals were amplified using a high-gain, low-noise amplifier (AU 1291, Miteq, Hauppage, NY, USA; gain, 63 dB) and demodulated in a dual homodyne-based IQ demodulator device (AD8333, Analog Devices, Norwood, MA, USA; RF frequency range, DC to 50 MHz). The demodulator also received a local oscillator reference signal from the function generator as input. The resulting I and Q signals were digitized using a 16-bit data acquisition (DAQ) card (PCIe 6363, National Instruments, Austin, TX, USA; maximum sampling rate per channel, 1 MS·s^−1^), which also controlled the galvanometric mirrors. The time that was required for a single-frequency scan of 300 × 300 pixels was ~0.6 s based on a dwell time per scan point of 4.2 µs, which was matched to the output filter of the IQ demodulator. The corresponding FOV could be adjusted by changing the voltage waveforms used for the galvanometric scanning unit. To increase the SNR, each image was generated by averaging sequential two-dimensional scans and correcting for heterogeneity in the transducer’s sensitivity field using a suitable two-dimensional Gaussian distribution. MATLAB (MATLAB 2014a, Mathworks, Natick, MA, USA) was used to control the microscopy system and process the images; volume rendering of the phantom samples was realized using Amira 3D software. Image post-processing, which involved gamma correction, Gaussian blurring, and windowing, was carried out in ImageJ (1.50e, Wayne Rasband).

### Multiphoton microscopy setup

Multiphoton excitation for generating SHG, THG, and TPEF signals was achieved using an Yb-based solid-state femtosecond laser oscillator that emitted at a central wavelength of 1043 nm (YBIX, Time-Bandwidth, Zurich, Switzerland; pulse width, 170 fs; average power output, 2.8 W; repetition rate, 84.4 MHz). The laser beam was initially attenuated using neutral density filters and subsequently guided onto the same set of galvanometric mirrors as for FDOM. To combine FDOM with multiphoton microscopy, we co-aligned the 808-nm beam with the 1043-nm beam using a short-pass dichroic mirror (DMSP1000, Thorlabs) and combined the 488-nm beam using a long-pass dichroic mirror (DMLP650, Thorlabs). Optoacoustic measurements were performed using a silver mirror to guide the 488 and 808 nm beams into the microscope, while a short-pass dichroic mirror (DMSP805R, Thorlabs) was used to guide the 1043 nm beam into the microscope to enable detection of SHG and TPEF signals in the backward direction. Appropriate narrow bandpass interference filters were used for selecting the desired SHG signals at ~522 nm (FB520–10) and TPEF emission (LP550), before the signals were recorded by an ultra-sensitive photomultiplier tube (H9305−03, Hamamatsu, Hamamatsu City, Japan). The primarily forward-directed THG signal was collected in transmission mode by employing an extra detection channel that consisted of an aspheric condenser lens (ACL25416U, Thorlabs; air immersion, NA 0.79), a UV-coated focusing lens (LA4052-UV, Thorlabs), a colorglass filter (FGUV5, Thorlabs) that was highly transparent to the detected THG UV wavelength of ~348 nm, and a second identical photomultiplier tube. The same 16-bit DAQ card, as was used for FDOM, was also used to digitize and acquire multiphoton signals and to control the galvanometric mirrors. Multiphoton devices were synchronized using a custom-designed LabVIEW (National Instruments) program and image processing was carried out in MATLAB and ImageJ.

### SNR comparison

A diode-pumped solid-state laser (SPOT-10–200–532, Elforlight Ltd, Daventry, UK) that emitted pulses of 532 nm light with 1.4 ns width at a repetition rate of 20 kHz was used for TD imaging^30^. The theoretical difference between FD and TD SNR values under the employed experimental conditions was calculated as follows: The TD pulse profile, denoted as $I_p\left( t \right),$ was assumed to follow a Gaussian shape^31^ with $I_p\left( t \right) \approx \phi _p{\mathrm{/}}\left( {\sigma _p\sqrt {2\pi } } \right) \cdot e^{ - t^2/2\sigma _p^2}$, whereas the FD laser intensity profile, denoted as $I_{CW}\left( t \right),$ can be expressed as $I_{CW}\left( t \right) = I_0\left[ {1 + \cos \left( {\omega _{mod}t} \right)} \right]$. Here, $\phi _p$ represents the optical fluence, $\sigma _p = t_p{\mathrm{/}}2\sqrt {2\,{\mathrm{ln}}(2)}$, *t*_*p*_ is the FWHM laser pulse length, $\omega _{mod}$ denotes the angular modulation frequency and *I*_*o*_ is the average light intensity. The optoacoustic pressure generated by a point source is proportional to the time derivative of the laser intensity profile. Therefore, the difference in optoacoustic pressure amplitude between the pulsed and CW excitations, in decibels, can be expressed as

1 $$\Delta p \approx 20\,log\left( {1.34\frac{{\phi _p}}{{t_p^2\,\omega _{mod}I_o}}} \right) \\ = 20\,log\left( {1.34\frac{{E_p}}{{t_p^2\,\omega _{mod}P_{CW}}}\frac{{\lambda _{cw}^2}}{{\lambda _p^2}}} \right)$$

where the factor 1.34 corresponds to $8\,{\mathrm{ln}}(2){\mathrm{/}}\sqrt {2\pi e}$, *E*_*p*_ denotes the energy per laser pulse, $\lambda _p$ and $\lambda _{cw}$ represent the wavelengths of the pulsed and CW excitations, respectively, and $P_{cw}$ denotes the average power of the CW laser. The right side of Eq. 1 takes into account that the same objective lens was used for both lasers and, thus, the respective values of the areas in $\phi _p$ and $I_o$ only differ in terms of the wavelengths. For *ω*_*mod*_= 2π × 50 MHz and the experimental laser settings used in the suture phantom measurements (pulsed laser: $E_p$ = 10 nJ, $\lambda _p$ = 532 nm, *t*_*p* _= 1.4 ns; CW laser: $P_{cw}$ = 18 mW, $\lambda _{cw}$ = 488 nm), the theoretical SNR is ~60.1 dB larger for pulsed than for CW excitation. Evaluating Eq. 1 using instead the respective ANSI limits for a pulsed excitation with 1.4-ns pulses in TDOM ($\phi _{p,\,ANSI}$ = 20 mJ·cm^−2^) and for a laser exposure time per pixel of 4.2 µs in FDOM ($I_{o,ANSI}$ = ~12 kW·cm^−2^) yields an SNR difference of ~70 dB.

Since the SNR is proportional to the square root of the detection bandwidth, the 90 kHz input bandwidth of the IQ demodulator compensates the SNR difference that is expressed by Eq. 1 by ~29 dB compared with the −3 dB bandwidth of the 75 MHz transducer of ~70 MHz used in TD imaging.

Furthermore, several modulation frequencies were used for each FDOM scan and subsequently summed, thereby resulting in another SNR compensation of

2 $${\mathrm{\Delta }}SNR_{Nmod} = \frac{1}{{\sqrt {N_{mod}} }} \cdot \mathop {\sum }\limits_{i = 1}^{N_{mod}} \frac{{f_i}}{{50\,{{\rm MHz}}}}$$

relative to the SNR difference that is expressed by Eq. 1, where a modulation frequency of 50 MHz was used. Here, *N*_*mod*_ is the number of modulation frequencies that were used and *f*_*i*_ denotes modulation frequency *i* in MHz. The sum in Eq. 2 accounts for the difference by a factor of $f_i{\mathrm{/}}50\,{\rm MHz}$ between the signal amplitude at each modulation frequency *f*_*i*_ and the signal amplitude at 50 MHz, while the noise is the same for all signals. For $N_{mod}$ = 9 and an equal distribution of nine frequencies between 10 MHz and 50 MHz, this results in an SNR compensation of ~5.1 dB. Taking into account that each final FD image is the average of 100 scans (20 dB), we estimate the theoretical SNR difference between FDOM and TDOM to be ~−6 dB, which is on the same order as the SNR difference that was experimentally derived from the suture images (Fig. 2g, h).

The deviation of the theoretical from the experimental SNR can be explained by the TD signals from the suture sample being broadband—spanning hundreds of MHz—whereas the transducer bandwidth is finite, and by the presence of acoustic attenuation. Thus, parts of the generated signals are located outside of the detection bandwidth, which reduces the achievable SNR in TDOM compared with the value obtained via Eq. 1. For the ~70-MHz bandwidth of the transducer and further considering the acoustic attenuation along the path of 3.6 mm in water, the aforementioned effects lead to another SNR reduction in TDOM of ~6 dB compared with FDOM. Other potential factors are a deviation in the optical focal plane between the FDOM and TDOM lasers, different noise figures of the acquisition electronics used in TDOM and FDOM and a deviation of the true pulse shape of the TDOM laser from an ideal Gaussian, as assumed in the derivation of Eq. 1.

### Doppler measurements

The sample was a suspension of glassy carbon particles (2–12-µm in diameter) in distilled water (volume fraction of ~20%). To avoid sedimentation, the mass density of the water was increased to match the density of the carbon particles (~1.45 g·cm^−^³) via the addition of an appropriate amount of sodium polytungstate^25^. Tween-20 was added to a final volume concentration of 1% to prevent particle aggregation. The particle suspension was pumped at known speeds through a microfluidic chip (product no. 01-0175-0138-02, microfluidic ChipShop, Jena, Germany; channel cross-section, 1 mm × 200 µm; lid, 140 µm TOPAS) using a syringe pump (540060, TSE systems, Bad Homburg, Germany).

The conically shaped 75-MHz transducer was mounted at an angle of ~55° with respect to the flow direction in the *xy*-plane, such as through vessels in the mouse ear. This configuration was chosen to achieve an appropriate Doppler angle for the optoacoustic Doppler shift *Δf*, which is described by the following equation:

3 $$\begin{array}{*{20}{c}} {\Delta f = f_{mod}\frac{{v_{abs}}}{c}\cos \left( \alpha \right)} \end{array}$$

Here, *f*_*mod*_ denotes the laser modulation frequency, *v*_*abs*_ is the absorber speed, *c* denotes the speed of sound in the medium, and *α* represents the Doppler angle. During galvanometric scanning, to provide high-resolution anatomical imaging, the transducer was positioned in positive defocus to allow for laser scanning within the sensitivity field, and the IQ demodulator was used for homodyne detection. For Doppler shift detection, a lock-in amplifier (UFHLI, Zurich Instruments, Zurich, Switzerland) was used as a spectrum analyzer. Initially, a static reference measurement of black polish was obtained. In the next step, Doppler shifts of carbon particles were measured as a function of flow speed. For each flow speed, 10 Doppler measurements were averaged and the resulting spectra were smoothed via locally weighted linear regression to enhance the signal visibility.

In the two-dimensional and profile measurements of blood flow, a modulation frequency of 42 MHz was chosen based on initial galvanometric scans to provide a high SNR for vasculature in the imaged region. For the two-dimensional flow scan, the sample was scanned in 8 µm steps in the *xy*-plane, while transducer and optical foci remained co-aligned and stationary. Signals were analyzed for Doppler shifts by applying a moving average filter to the raw signals and fitting a double-Gaussian function to the flow distribution. The prominent central peak at *f* *=* *f*_*mod*_ was removed and interpolated for this analysis. If the peak amplitude of the fit fell below a certain threshold value or if the fit quality was poor, the respective pixels in the image were set to zero. For the profile scanning of blood flow, the step size was 2 µm. In the spectrum that corresponds to a lateral position of 14 µm in Fig. 4k, the frequency range of −15 to −5 Hz was excluded from the fit, as it was identified as noise that was distinct from the flow peak.

### IQ demodulation

We used an IQ-demodulator with a 50 MHz bandwidth (Model AD8333, Analog Devices) for fast, memory-efficient optoacoustic signal detection in the FD. The IQ demodulator enables homodyne detection of baseband signals that consist of an in-phase component $I\left( t \right) = A\cos \left[ {\varphi \left( t \right)} \right]$ and a quadrature component $Q\left( t \right) = A\sin \left[ {\varphi \left( t \right)} \right]$. Signals with amplitude *A* and phase *φ* were extracted from acquired *I* and *Q* data using the following equations:

4 $$A = \sqrt {{I}^2 + {Q}^2}$$

5 $${\varphi } = {\tan}^{ - 1}\left( {\frac{{Q}}{{I}}} \right)$$

Demodulated signals passed through a 90 kHz low-pass filter. For multispectral measurements at 488 nm and 808 nm, the signal from the transducer was amplified and split into two separate IQ demodulators. After mixing with the corresponding local oscillator signals, IQ signals for each wavelength were recorded using the DAQ.

## Electronic supplementary material

Supplementary Material
