# Supplementary material for: Optoacoustic microscopy at multiple discrete frequencies
Source: Light Sci Appl. 2018 Dec 19;7:109. doi: 10.1038/s41377-018-0101-2 (PMC6298999; doi:10.1038/s41377-018-0101-2)
Supplement: Supplementary file 1 — Supplementary Material [file 41377_2018_101_MOESM1_ESM.docx]

SUPPLEMENTARY MATERIAL for

Optoacoustic microscopy at multiple discrete frequencies

Stephan Kellnberger^1,2,3†^, Dominik Soliman^1,2†^, George J. Tserevelakis^1,2†^, Markus Seeger^1,2†^, Hong Yang^1,2,4^, Angelos Karlas^1,2^, Ludwig Prade^1,2^, Murad Omar^1,2^, Vasilis Ntziachristos^1,2^*

^1^Institute of Biological and Medical Imaging (IBMI), Helmholtz Zentrum München, Ingolstädter Landstr. 1, 85764 Neuherberg, Germany.

^2^Chair of Biological Imaging (CBI) and Center for Translational Cancer Research (TranslaTUM), Technical University Munich, Einsteinstraße 25, 81675 München, Germany.

^3^Cardiovascular Research Center, Cardiology Division, Massachusetts General Hospital, Harvard Medical School, Boston, Massachusetts 02114, USA.

^4^Xidian University, South Taibai Road 02, 710071, Xi’an, Shaanxi, China.

^†^These authors contributed equally to this work

*Corresponding author: v.ntziachristos@tum.de

**SUPPLEMENTARY FIGURES**

**
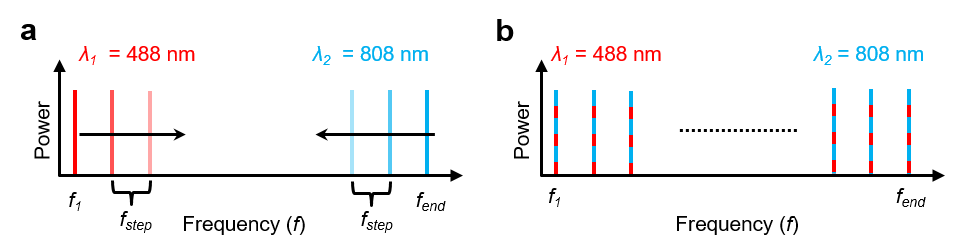
**

**Supplementary Figure S1 | Explanation of frequency coding in dual wavelengths FDOM. a** Simplified schematic of frequency coding on different wavelengths. Laser source 1 emitting at *λ_1_ =* 488 nm was loaded with the lowest modulation frequency *f_1_*, while laser source 2 emitting at *λ_2_ =* 808 nm was loaded with the highest modulation frequency *f_end_*. During imaging, we increased the modulation of wavelength *λ_1_* and decreased the modulation frequency of *λ_2_* in steps of *f_step_* using odd numbers of modulation frequencies. **b** Schematic representation of multiple modulation frequencies used for imaging, showing the superposition of frequencies at two wavelengths.


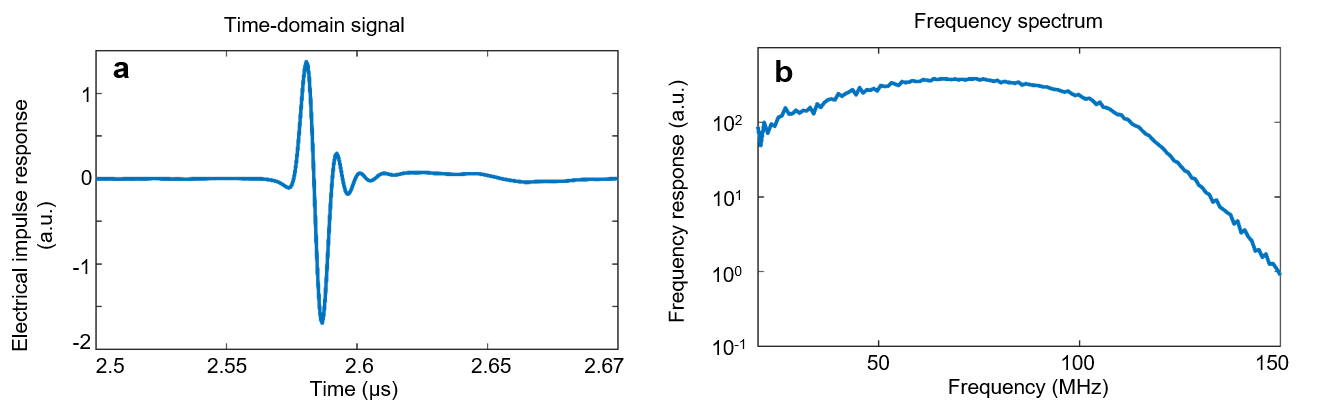


**Supplementary Figure S2 | Frequency response of the transducer. a** Time-domain representation of the electrical frequency response of the transducer, detected from an optoacoustic point source**. b** FFT representation of the electrical frequency response, showing a -12 dB bandwidth of 117 MHz and a central frequency of 75 MHz.


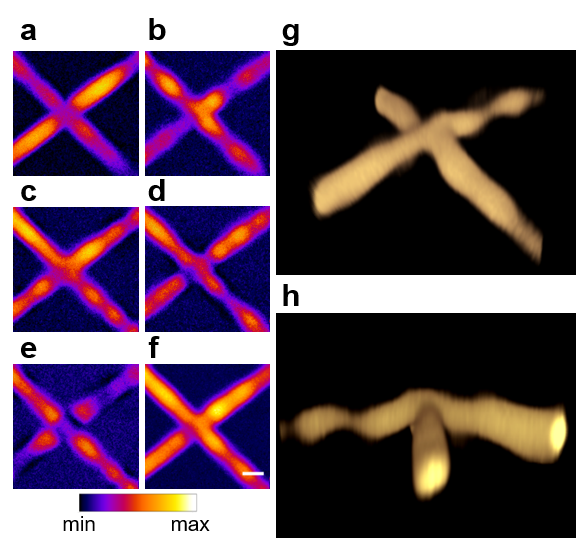


**Supplementary Figure S3 | FDOM imaging of two crossing sutures (50 µm diameter) at different modulation frequencies.** **a** FSR at 30 MHz. **b** FSR at 35 MHz. **c** FSR at 40 MHz. **d**FSR at 45 MHz. **e** FSR at 50 MHz. **f** Image reconstruction based on time-space representation (TSR) using all frequencies. Scale bar, 50 µm. **g, h** Three-dimensional TSR-based reconstruction showing the three-dimensional volume of the reconstructed sutures in isometric and side views.


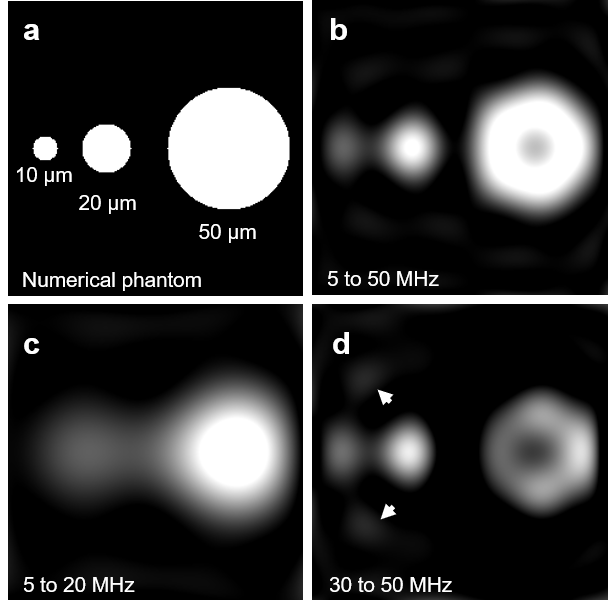


**Supplementary Figure S4 | Simulation study showing the effect of modulation frequencies on image reconstruction. a** True absorption image of the phantom containing 3 absorbers. **b**Reconstruction result based on eight evenly spaced frequencies between 5 and 50 MHz. **c** Reconstructed image based on the eight evenly spaced frequencies between 5 and 20 MHz. **d**Reconstruction using eight evenly spaced frequencies between 30 and 50 MHz. Arrows denote spatial frequency artifacts due to an unmatched selection of modulation frequencies.
This finding implies that conventional TD optoacoustics based on broadband stimulation substantially oversamples frequencies, indicating an information acquisition redundancy.


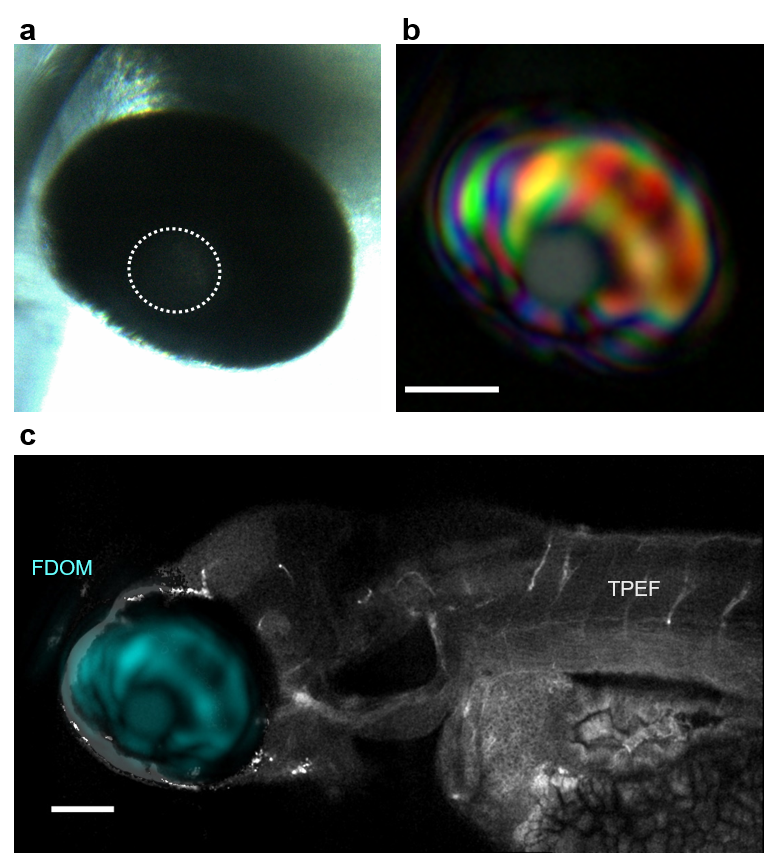


**Supplementary Figure S5 | Hybrid FDOM (808 nm) and two-photon excitation fluorescence (TPEF) imaging of an 8-day-old transgenic Casper-type zebrafish larva *ex vivo*. a** Bright-field image of the eyeball. The highlighted circle indicates the lens. **b** Multi-frequency RGB image of the eyeball obtained using modulation frequencies from 30 to 50 MHz. Red depicts the amplitude contribution of the modulation frequency *f_mod_* = 30 MHz; green, the contribution of 40 MHz; and blue, the contribution of 50 MHz. **c** Superposition of 808 nm FDOM and TPEF images of the zebrafish. Cyan color shows the result of summing the amplitudes of the three frequency components shown in panel **b**; gray color shows TPEF of GFP from the fish body. Scale bar, 100 µm.


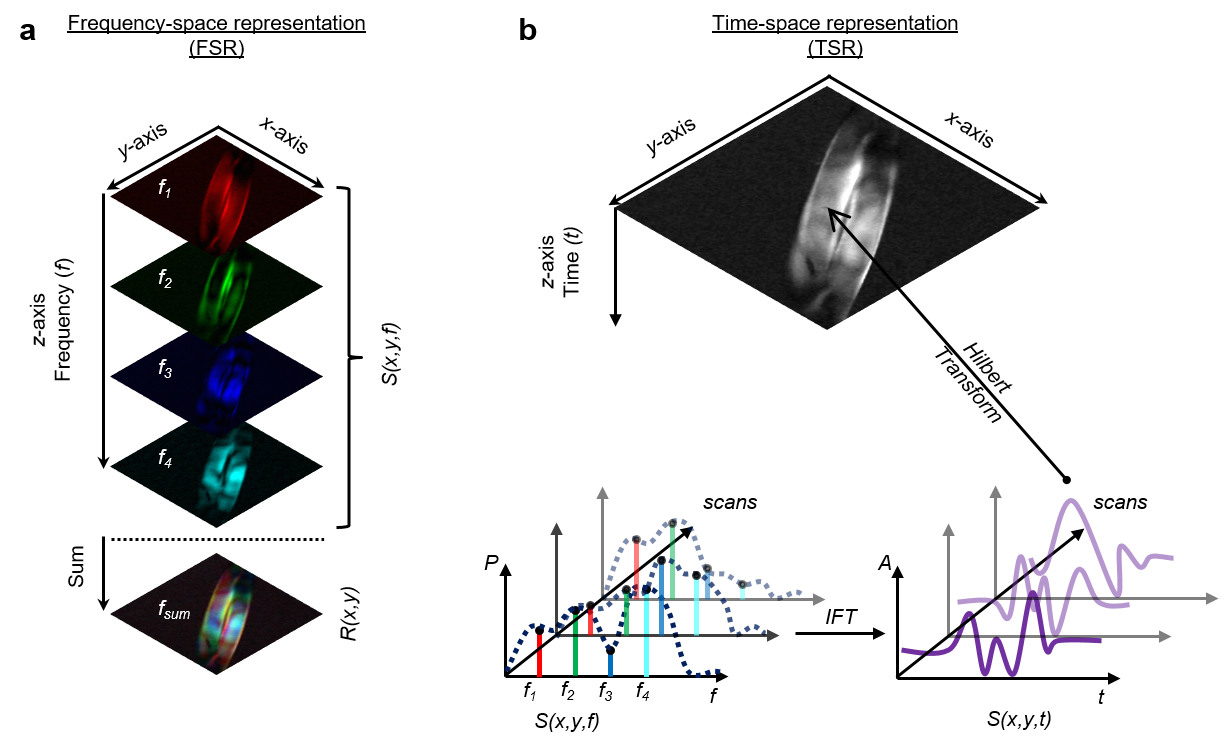


**Supplementary Figure S6 | Simplified schematic illustrating image formation using frequency-space representation (FSR) or time-space representation (TSR). a** In FSR image formation, the image is reconstructed based on the summation of selected frequencies according to $\text{Recon}_{FSR}\left(x,y,\sum_{i=1}^{n} f\right)$. In this example, the three-dimensional data set *S(x,y,f),* in which *x* and *y* correspond to the raster scanning points and *f* corresponds to the respective modulation frequency, is projected onto a two-dimensional map *R(x,y)*. The final image represents the sum of all spatial frequency components. **b** In TSR image formation, the three-dimensional data set *S(x,y,f)* is converted to the time domain using inverse Fourier transformation (IFT), yielding the time-dependent three-dimensional matrix *S(x,y,t).* This matrix is subjected to a Hilbert transformation to generate the FDOM image.


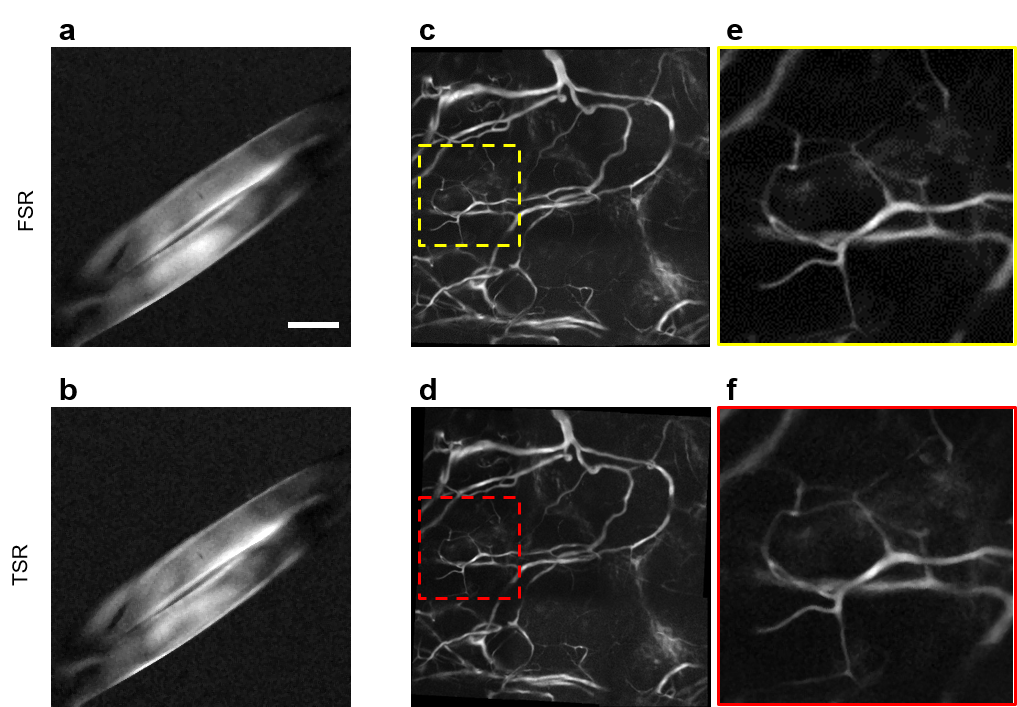


**Supplementary Figure S7 | Comparison of TSR- and FSR-based imaging of suture phantoms and *in vivo* samples.** **a** FSR-based reconstruction of two sutures (50 µm diameter) at modulation frequencies of 10, 20, 30, and 40 MHz. Scale bar, 50 µm. **b** TSR-based reconstruction of the same data set as in panel **a** at the same four modulation frequencies. **c** FSR-based reconstruction of mouse ear *in vivo* using nine modulation frequencies between 10 MHz and 50 MHz. **d** TSR-based reconstruction of the same data set as in panel **c** at the same nine modulation frequencies. **e** Close-up view of the highlighted area in panel **c**. **f** Close-up view of the highlighted area in the TSR image in panel **d**, showing similar contrast and SNR as in the FSR-based reconstruction in panel **e**.


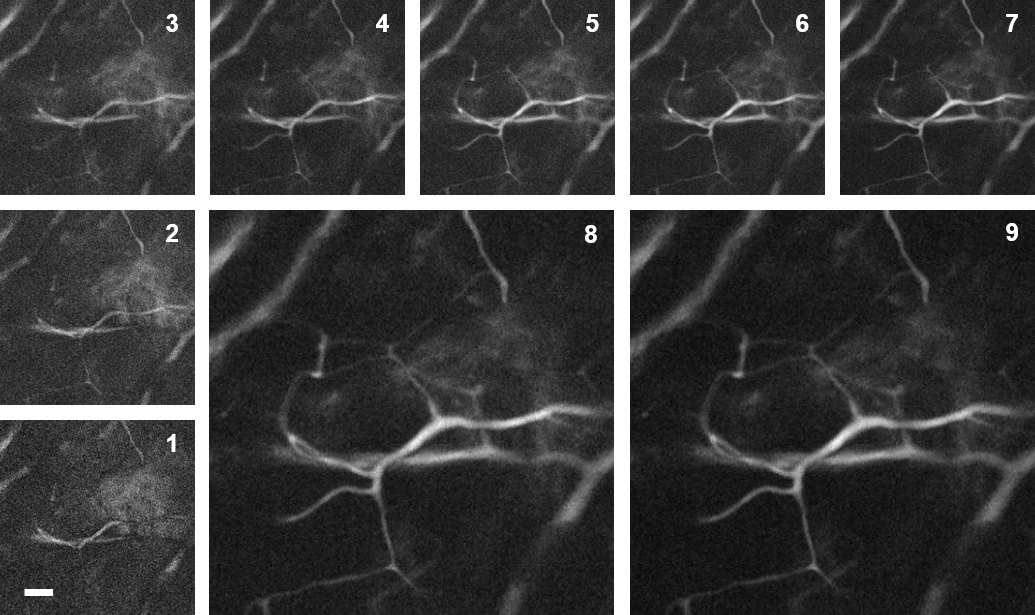


**Supplementary Figure S8 | Signal-to-noise ratio (SNR) following FSR-based reconstruction as a function of modulation frequencies.** FSR-based reconstructions $\text{Recon}_{FSR}\left(x,y,\sum_{i=1}^{n} f\right)$ were performed according to nine scenarios involving different numbers and ranges of modulation frequencies *f*. Scenario 1 (*n* = 1): 10 MHz. Scenario 2 (*n* = 2): 10 and 15 MHz. Scenario 3 (*n* = 3): 10, 15, and 20 MHz. Scenario 4 (*n* = 4): 10, 15, …, 25 MHz. Scenario 5 (*n* = 5): 10, 15, …, 30 MHz. Scenario 6 (*n* = 6): 10, 15, …, 35 MHz. Scenario 7 (*n* = 7): 10, 15, …, 40 MHz. Scenario 8 (*n* = 8): 10, 15, …, 45 MHz. Scenario 9 (*n* = 9): 10, 15, …, 50 MHz. Scale bar, 50 µm.


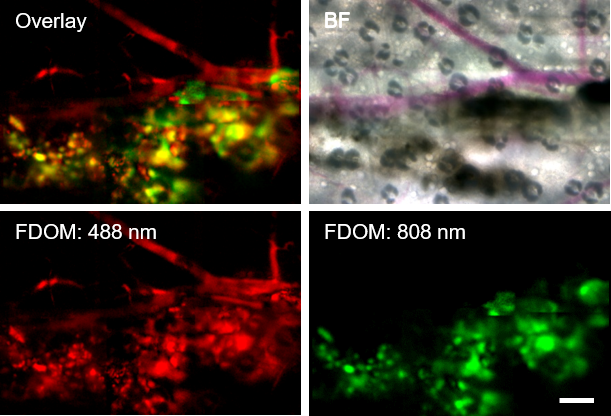


**Supplementary Figure S9 | Hybrid FDOM and multiphoton microscopy of a mouse ear *ex vivo*. FDOM imaging of blood vessels in a CD1 mouse ear *ex vivo*.** At 24 hours before imaging, B16F10 melanoma cells were injected directly into the ear *in vivo*. FDOM imaging at 488 nm (red) and seven modulation frequencies from 10 to 40 MHz reveals vasculature as well as melanoma cells; in contrast, FDOM imaging at 808 nm (green) and the same seven modulation frequencies reveals only melanoma cells. The bright-field (BF) image validates the FDOM results, showing vasculature and injected melanoma cells. The overlay image shows vasculature based on FDOM at 488 nm and melanoma cells based on FDOM at 808 nm. Scale bar, 200 µm.


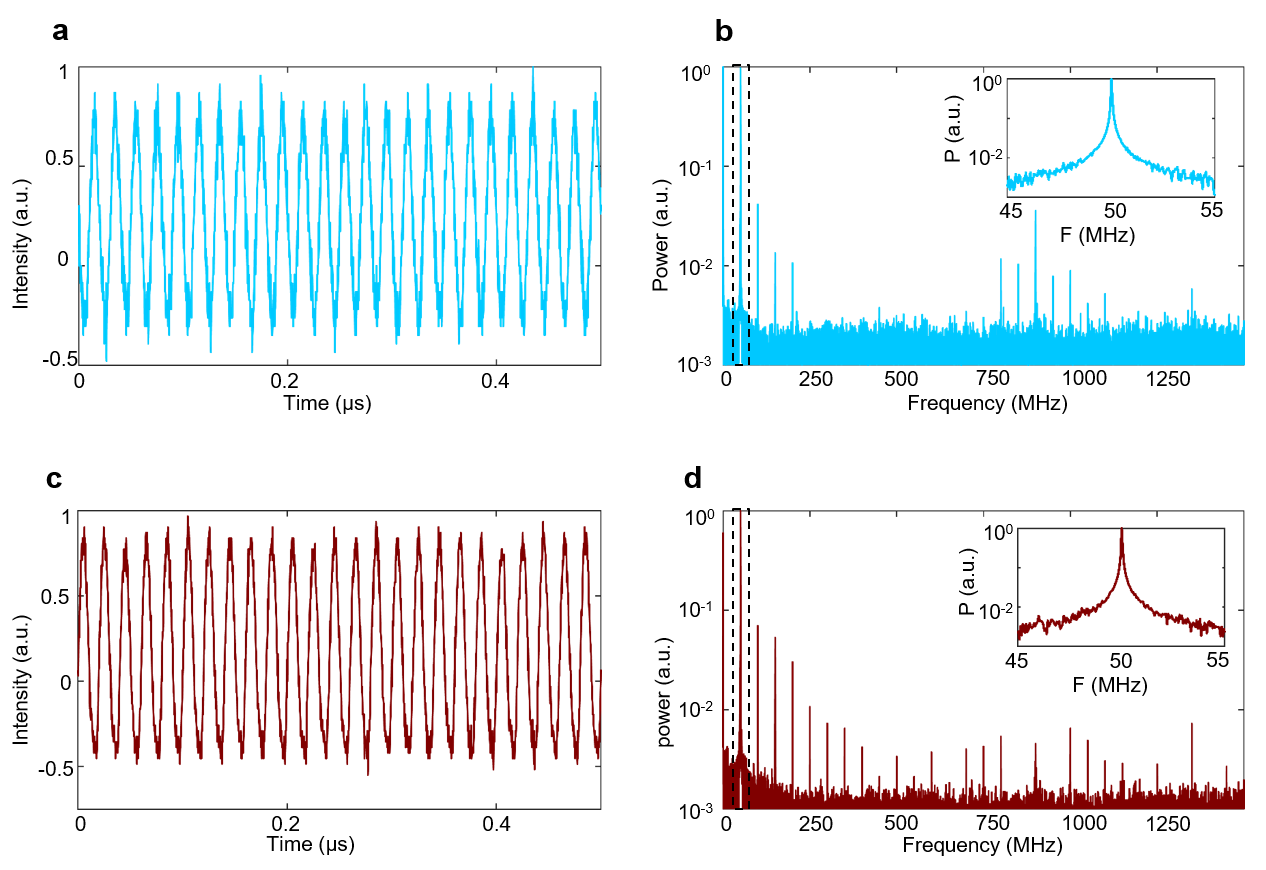


**Supplementary Figure S10 |** **Laser outputs of the 488 nm and 808 nm laser unit, acquired at 50 MHz sine-wave modulation. a** Sine wave of the 488 nm laser module as a function of time. **b**Frequency spectrum of **a**, showing higher harmonics. The inset illustrates the 50 MHz modulation component. **c** Sine wave modulation of the 808 nm laser module as a function of time. **d** Frequency spectrum of **c**, depicting higher harmonics due to an imperfect sine wave signal. The inset shows a close-up view of the 50 MHz component.


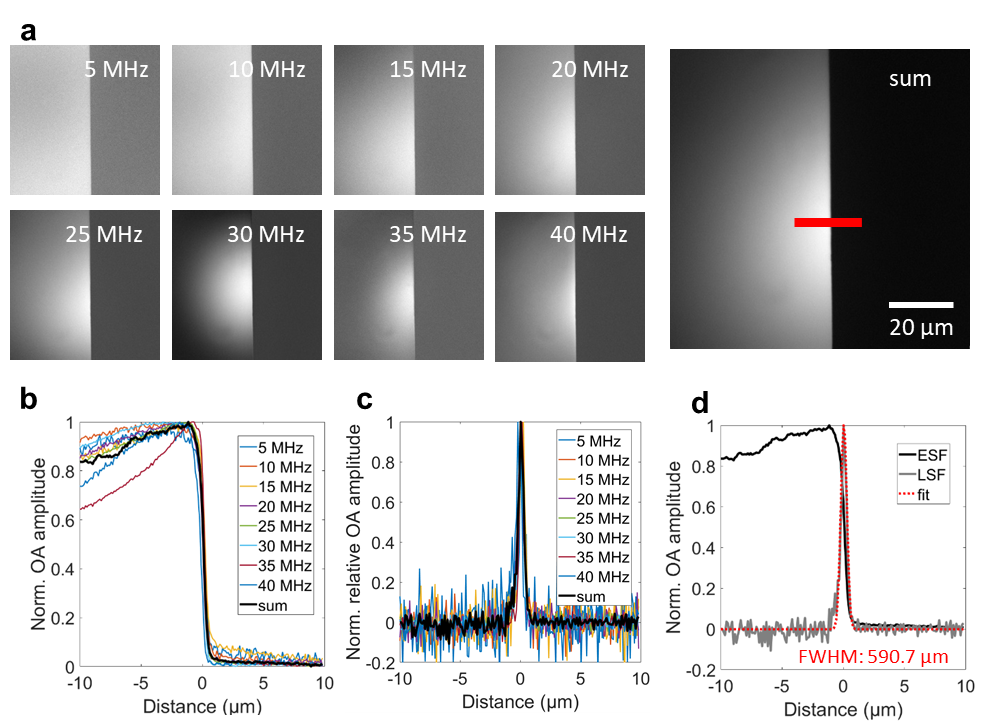


**Supplementary Figure S11 | Razor edge measurement to determine the lateral resolution of the FDOM system at 488 nm. a** FSR images at different frequencies from 5 to 40 MHz in 5 MHz steps and the *z*-projected image. **b**Edge spread function (ESF) at different frequencies. **c** Line spread function (LSF), showing the FWHM of the laser spot as a function of modulation frequency. **d**ESF, LSF, and a Gaussian fit to determine the lateral resolution of 590.7 µm of the FDOM system based on the sum of eight frequencies from 5 to 40 MHz.


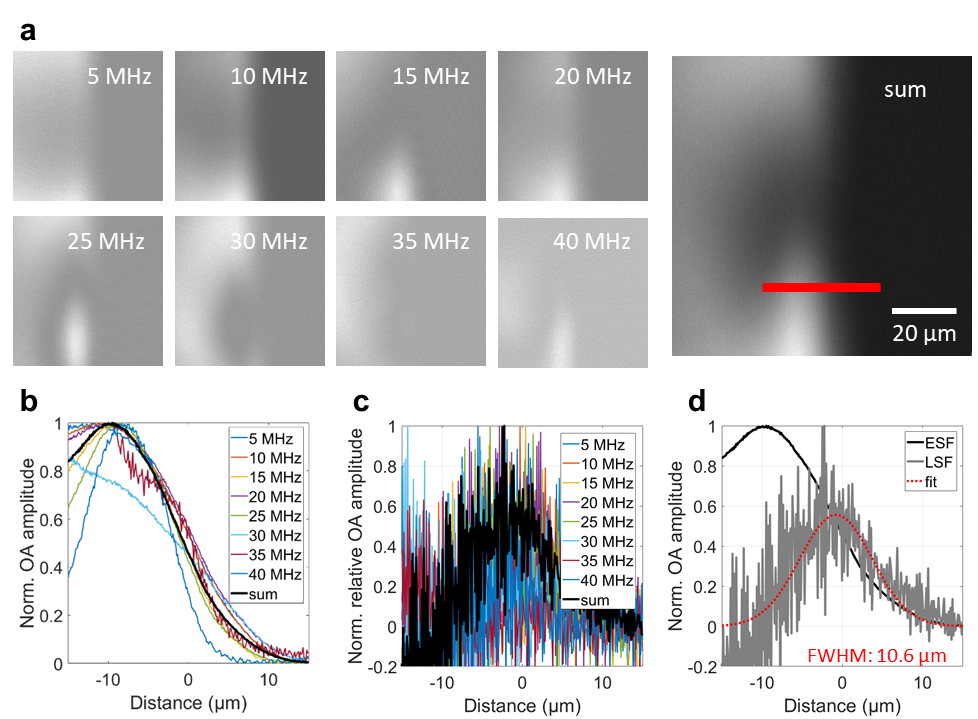


**Supplementary Figure S12 | Razor edge measurement to determine the lateral resolution of the FDOM system at 808 nm. a** FSR images at different frequencies from 5 to 40 MHz in 5 MHz steps and the *z*-projected image. **b**Edge spread function (ESF) at different frequencies. **c** Line spread function (LSF), showing the FWHM of the laser spot as a function of modulation frequency. **d**ESF, LSF, and a Gaussian fit to determine the lateral resolution of 10.6 µm of the FDOM system based on the sum of eight frequencies from 5 to 40 MHz.
